# The influence of pacific winds on ENSO diversity

**DOI:** 10.1038/s41598-021-97963-4

**Published:** 2021-09-21

**Authors:** Antonietta Capotondi, Lucrezia Ricciardulli

**Affiliations:** 1 Cooperative Institute for Research in Environmental Sciences, University of Colorado, Boulder, CO USA; 2 NOAA/Physical Sciences Laboratory, 325 Broadway, Boulder, CO 80305 USA; 3 Remote Sensing Systems, Santa Rosa, CA USA

**Keywords:** Climate sciences, Ocean sciences

## Abstract

The differences in ENSO sea surface temperature (SST) spatial patterns, whether centered in the Eastern Pacific (EP), Central Pacific (CP) or in the eastern-central equatorial region (“canonical”) have been associated to differences in atmospheric teleconnections and global impacts. However, predicting different types of ENSO events has proved challenging, highlighting the need for a deeper understanding of their predictability. Given the key role played by wind variations in the development and evolution of ENSO events, this study examines the relationship between the leading modes of Pacific surface wind speed variability and ENSO diversity using three different state-of-the-art wind products, including satellite observations and atmospheric reanalyses. Although previous studies have associated different ENSO precursors to either EP or CP events, our results indicate that the most prominent of those ENSO precursors are primarily related to canonical and CP events, and show little correlation with EP events. The latter are associated with tropical Pacific conditions favoring equatorial westerly wind and precipitation anomalies that extend all the way to the eastern Pacific. Results over the entire twentieth century period versus those during the satellite era also suggest that the influences from the Southern Hemisphere may be more robust than those from the Northern Hemisphere.

## Introduction

The El Niño Southern Oscillation (ENSO) is the dominant mode of climate variability at interannual timescales in the tropical Pacific, with very important influences worldwide. Surface winds play a key role in the initiation, growth and demise of ENSO events through both a deterministic wind component arising as a response to sea surface temperature (SST) anomalies^1^ as well as a stochastic component (Capotondi et al.^2^, and references therein).

ENSO events differ in their amplitude and spatial patterns as emphasized by recent literature^3,4^. These differences range from the extreme El Niño event of 1997/1998, which had the largest anomalies in the Eastern Pacific to the weaker events during 2000–2010 with peak anomalies around the dateline. These differences in spatial patterns, which are more pronounced for the warm (El Niño) phase of ENSO than for the cold La Niña conditions^5^, are associated with different atmospheric teleconnections and large-scale impacts^6^. For example, while ENSO events are generally viewed as the primary source of predictability for ocean conditions in the California Current System, with important implications for marine ecosystems in that region^7^, it is the SST anomalies in the central/western equatorial Pacific, like those associated with Central Pacific (CP) ENSO events, that are more influential on SST anomalies along the US West Coast^8,9^. SST anomalies in the Eastern equatorial Pacific (as in EP ENSO events), on the other hand, may be more influential on precipitation over California^9^. Thus, the ability to predict different ENSO types is critical, although, to-date, it has proved very challenging^10,11^.

Influences from the extra-tropical Pacific have been proposed as possible sources of ENSO predictability. The most prominent of these extra-tropical precursors include the Pacific Meridional Mode (PMM)^12^, the Western North Pacific Precursor (WNP)^13^, and the South Pacific Meridional Mode (SPMM)^14^. These ENSO precursors have primarily been described in terms of SST anomalies: the PMM is characterized by SST anomalies that extend southwestward from the coast of California to the central equatorial Pacific^15^; the WNP precursor is defined in terms of SST anomalies in the western North Pacific region (122°–132° E to 18°–28° N)^13^; and the SPMM exhibits anomalies extending along the west coast of South America toward the eastern equatorial Pacific^14^. However, these precursors also include important sea level pressure (SLP) and wind signatures, which can influence ENSO events through both thermo-dynamical (e.g., changes in turbulent heat fluxes) and dynamical (e.g., excitation of oceanic waves and changes in ocean advection) processes^15^. These various precursors have been associated with different types of El Niño events: the PMM has been viewed as a precursor for CP events^16,17^, while the WNP and SPMM have been considered more influential on EP El Niño events^17,18^. Other studies, however, have emphasized the key discriminating role of the initial/background subsurface ocean conditions, which may themselves result from large-scale wind patterns, for the development of different El Niño types^19,20^.

These extra-tropical ENSO precursors are not necessarily independent, but they are likely linked by the large-scale atmospheric circulation, as captured by the basin-scale wind field. Thus, large-scale patterns of wind variations could be used to examine extra-tropical influences on ENSO and its diversity. In situ wind observations from moored buoys provide an invaluable “ground truth” where available (e.g., the TAO/TRITON array in the Pacific^21^), but the large-scale fields needed for climate studies are usually obtained from atmospheric reanalysis products. These, however, may assimilate different observations with different assimilation techniques, use different SST fields as lower boundary conditions, and suffer from model errors, factors that can all introduce inaccuracies and discrepancies among the different data sets^22^. Satellite data provide large-scale wind observations at a relatively high spatial and temporal resolutions, and are now of sufficient duration and accuracy to allow their utilization for climate studies^23,24^. In particular, wind speed has been continuously measured from space since 1988, and carefully inter-calibrated across different satellite missions^25^, providing an excellent data set for examining ENSO precursors. In this study, we will use these satellite-based wind speed data, complemented by those obtained from two state-of-the-art atmospheric reanalysis products to pursue the following objectives: (1) identify the dominant patterns of wind speed variability in the Pacific basin, their relationship with known ENSO precursors and their association with ENSO diversity; and (2) assess the robustness of these relationships across different products and time periods.

## Methods

### Datasets

We use three different wind speed (WS) datasets at monthly time resolution: (1) satellite ocean surface winds, from the 1° monthly merged radiometers climate data record available from 1988 to present (referred to as SAT in the graphics); (2) the ECMWF-ERA5 atmospheric reanalysis (0.5° horizontal resolution, 1979–2018)^26^; and (3) the NOAA-20CRv3 atmospheric reanalysis (~ 0.7° horizontal resolution, 1836–2015)^27^. These are very different wind products. The first is an observational dataset obtained by merging satellite microwave retrievals from several inter-calibrated radiometers (SSM/I, SSMIS, TMI, GMI, AMSR2, ASMR-E, WindSat), the second is an atmospheric reanalysis that assimilates a broad range of observations, and the third is a 179-year atmospheric reanalysis that only assimilates SLP, in addition to prescribing SST from the Simple Ocean Data Assimilation with sparse input version 3 (SODAsi.3^28^) and HadISST2.2^29^, and prescribing sea ice concentration from HadISST2.3^30^, as lower boundary conditions^27^. The latter reanalysis is used for comparison with the two other products over the overlapping period 1988–2015 (20CR-1988 hereafter) as well as during 1900–2015 (20CR-1900 hereafter) to examine the robustness of the results over a century-long period. To more fully characterize the atmospheric conditions associated with the leading wind speed patterns we also use SLP, vector winds and precipitation, which are available from both atmospheric reanalyses. Since ECMWF-ERA5 and NOAA-20CRv3 are ensembles (10 and 80 members, respectively), we use the ensemble mean wind speed, which is computed from sub-daily values of each ensemble member. Our SST dataset is the Extended Reconstructed SST version 5 (ERSSTv5)^31^, and sea surface height (SSH), a quantity dynamically-related to upper-ocean heat content, is obtained from the ECMWF Ocean Reanalysis System 5 (ORAS5)^32^ available to us from 1979 to 2017.

### Statistical significance testing

This study makes use of correlation and regression analysis, whose statistical significance is assessed using a two-tailed student-t test. To account for the temporal autocorrelation of the time series, we computed an effective number of degrees of freedom N* following Bretherton et al.^33^, as:

1 $${N}^{*}=N\frac{(1-{r}_{1}{r}_{2})}{(1+{r}_{1}{r}_{2})},$$

where *N* is the total sample size, and *r*_1_ and *r*_2_ are the lag-1 autocorrelations for each of the time series.

### Indices of ENSO diversity

Several indices have been proposed to identify different types of ENSO events^4^. Here, we characterize ENSO diversity using the indices proposed by Takahashi et al.^34^. This approach relies on the two leading empirical orthogonal functions (EOFs) of SST anomalies in the equatorial Pacific (10° S–10° N), which explain a large fraction (~ 83%) of the SST variance in this region. The leading EOF exhibits a “canonical” ENSO pattern^35^, with positive anomalies that achieve their largest values in the central-eastern Pacific, while the second EOF displays a dipole pattern along the equator, with negative anomalies in the eastern Pacific, and positive anomalies in the central Pacific (Fig. S1). The time series, or Principal Component (PC) associated with the leading SST EOF (PC1) is used here as the index describing canonical ENSO events. Indices for EP and CP events are obtained as linear combinations of PC1 and PC2 divided by the square root of their corresponding eigenvalues λ_1_ and λ_2_, which represent their respective variances^17^:

2 $$EP = {{\left( {\frac{{PC1}}{{\sqrt {\lambda _{1} } }} - \frac{{PC2}}{{\sqrt {\lambda _{2} } }}} \right)} \mathord{\left/ {\vphantom {{\frac{{PC1}}{{\sqrt {\lambda _{1} } }} - \frac{{PC2}}{{\sqrt {\lambda _{2} } }}} {\sqrt 2 }}} \right. \kern-\nulldelimiterspace} {\sqrt 2 }},CP = {{\left( {\frac{{PC1}}{{\sqrt {\lambda _{1} } }} + \frac{{PC2}}{{\sqrt {\lambda _{2} } }}} \right)} \mathord{\left/ {\vphantom {{\left( {\frac{{PC1}}{{\sqrt {\lambda _{1} } }} + \frac{{PC2}}{{\sqrt {\lambda _{2} } }}} \right)} {\sqrt 2 }}} \right. \kern-\nulldelimiterspace} {\sqrt 2 }}$$

### Determination of precursors of ENSO diversity

To determine the basin-wide patterns of SST and SSH that are most conducive to either EP or CP events (“sensitivity patterns”) at some later time we use the same approach of Capotondi and Sardeshmukh^20^ and Capotondi et al.^8^. This approach relates each of the ENSO indices (*y*) at time *t*_*o*_ + *t* to the Pacific conditions at the initial time *t*_*o*_, as indicated by the state vector ***x***, through the operator ***H*** such that:

3 $$y\left(t+{t}_{o}\right)={\varvec{H}}(t) {\varvec{x}}({t}_{o})$$

The state vector ***x*** includes SST and SSH information. The inclusion of SSH was shown to be essential for capturing ENSO diversity^20^. The SST and SSH fields are smoothed with a 3-month running mean and linearly detrended before projecting them onto their leading EOFs, a step adopted to reduce the number of degrees of freedom. We retain 20 SST EOFs and 20 SSH EOFs, accounting for 80% and 66% of the corresponding field variance. ***H***(*t*) is computed through multiple linear regressions, and the initial state most conducive to *y* (*t* + *t*_*o*_) is determined as the leading right singular vector of ***H***.

## Results

### Patterns of wind speed variability

**Figure 1:**
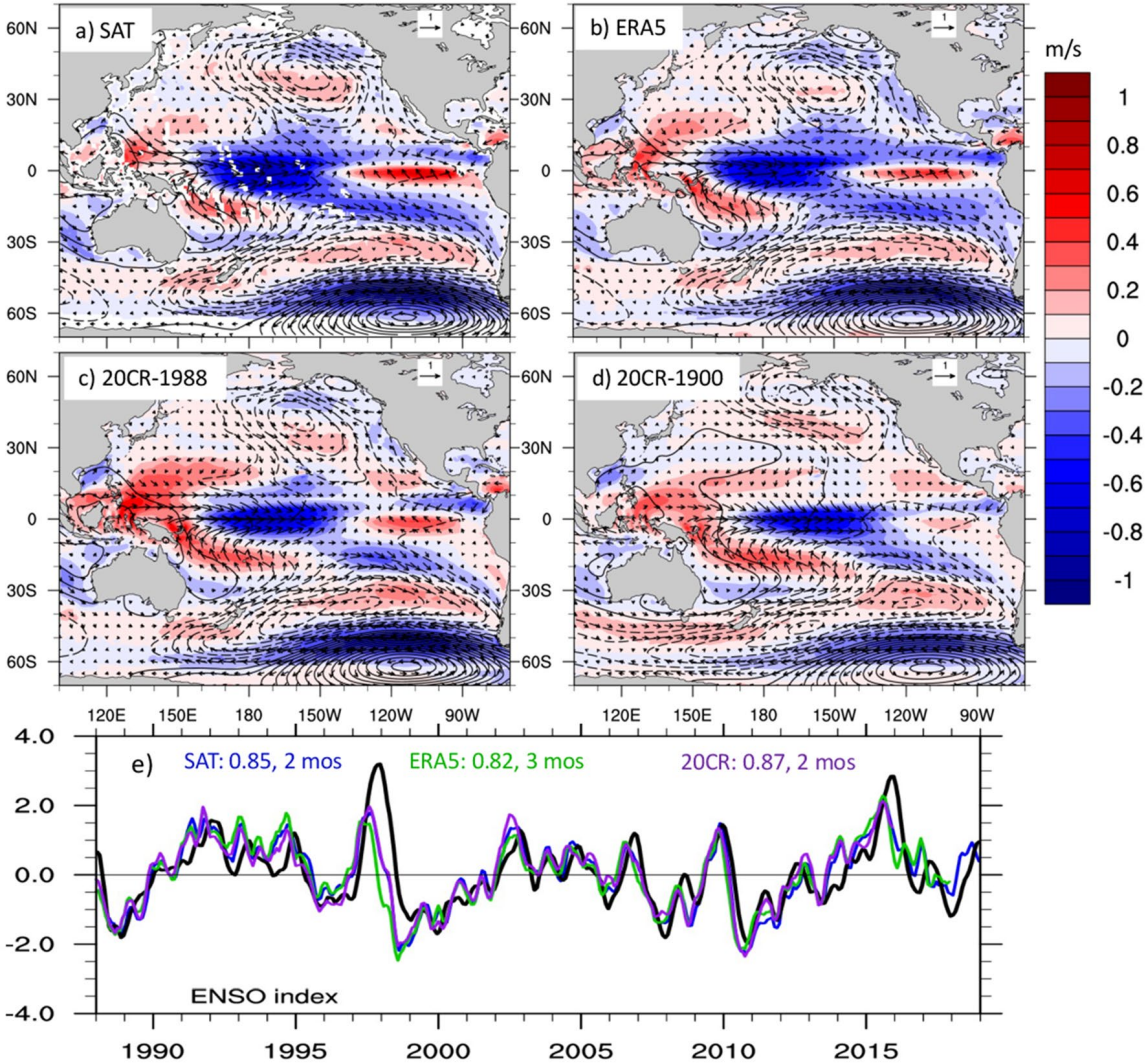
Pacific patterns of wind speed variability and relationship with ENSO. Leading EOF of wind speed for (**a**) Merged satellite winds (variance explained 14.3%), (**b**) ERA5 (12.8%), (**c**) 20CRv3 during 1988–2015 (12.8%), and (**d**) 20CRv3 over 1900–2015 (9%) over the Pacific basin (70° S–70° N, 100° E–70° W). Black contours show the regression of SLP anomalies (in Pa) upon the EOFs’ time series, or Principal Components (PCs). Dashed contours indicate negative values. Arrows show the vector winds regressed on the leading PCs of each wind speed product. SLP and winds from ERA5 are used in (**a**) and (**b**), while SLP and winds from 20CRv3 are used in (**c**) and (**d**). Vector winds are in m/s. (**e**) Comparison between the canonical ENSO index (black, see “Methods”), and the PCs of the leading wind speed EOFs, as indicated by the different colors. Numbers on the top indicate the maximum correlation and the wind speed lead time at which the maximum correlation is achieved for each data set.

We start by examining the dominant patterns of wind speed variability over the Pacific basin (70° S–70° N, 120° E–90° W) using EOF analysis. Wind speed anomalies have been smoothed with a 3-month running mean and linearly detrended prior to the EOF calculation. The leading patterns are shown in Fig. 1a–c for merged-satellites, ERA5 and 20CR-1988, to directly compare the three different wind products over a largely overlapping recent period, and in Fig. 1d for 20CR-1900. The latter is intended to show the comparison with the leading EOF pattern over a much longer time period. The leading EOFs are very similar among the three different wind datasets, including during 1900–2015. The fraction of variance accounted for by this leading EOF is also similar for the three wind speed datasets, especially after 1988, with satellite winds explaining the largest fraction and 20CR-1900 the lowest fraction. Although the variance explained over the whole domain is not very large, this leading EOF is statistically separated from the higher modes, based on the criterion of North et al.^36^, where the number of independent realizations is estimated as outlined by Quadrelli et al.^37^. This leading pattern of variability can account for larger fractions of variance locally. For example, since 1988, this pattern explains more than 20% of the variance around 20° S and 50°–60° S in all three datasets, and as much as 40% and 60% of the variance in the eastern and central-western equatorial Pacific, respectively. The spatial patterns of EOF2 and EOF3 for the different datasets are displayed in Figs. S2 and S3 for comparison.

To understand the connection between these patterns of wind speed variability and atmospheric circulation, we regress SLP and vector winds on the standardized principal components (PCs) associated with the patterns in Fig. 1a–d, and overlay these fields on the wind speed patterns. The PCs are obtained by projecting the original wind speed anomalies onto the EOF patterns, and describe the temporal evolution of those patterns. Along the equator, the wind speed anomalies are associated with westerly wind anomalies in the central-western Pacific and easterly wind anomalies in the eastern part of the basin, consistent with the sign of the wind speed anomalies. In the Southern Hemisphere, SLP anomalies are characterized by a meridional dipole with negative and positive centers around 45° and 65° S, respectively. This dipole structure is very similar to the Pacific South American pattern 1 (PSA-1), which is defined as the second leading mode of 500-hPa geopotential height over the Southern Hemisphere^38,39^. A similar SLP structure, obtained through EOF analysis over a region in the eastern South Pacific, has been termed South Pacific Oscillation (SPO)^40^. In the Northern Hemisphere, SLP anomalies associated with our leading WS mode, exhibit a negative SLP center around 40° N, 160° W, which is consistent with a deepening and eastward extension of the Aleutian Low. These SLP anomalies are associated with a weakening of the off-equatorial trade winds and a strengthening of the far-western Pacific equatorward winds in both Hemispheres.

The leading PCs of wind speed show a very good correspondence with the canonical ENSO index (Fig. 1e) with correlation coefficients larger than 0.8 at a lead time of 2–3 months. While these lead times are somewhat consistent with the SPO being a boreal-summer ENSO precursor^40^, and with the lag between sub-seasonal equatorial wind variations and the ENSO peak^2^, the deepening of the Aleutian Low in the Northern Hemisphere is more typical of the mature phase of ENSO events. Since wind variations in the Northern Hemisphere typically peak during the boreal winter-spring season^15^, unlike the Southern Hemisphere winds^40^, the EOF calculation over the entire Pacific basin may confound the different hemispheric seasonalities.

**Figure 2:**
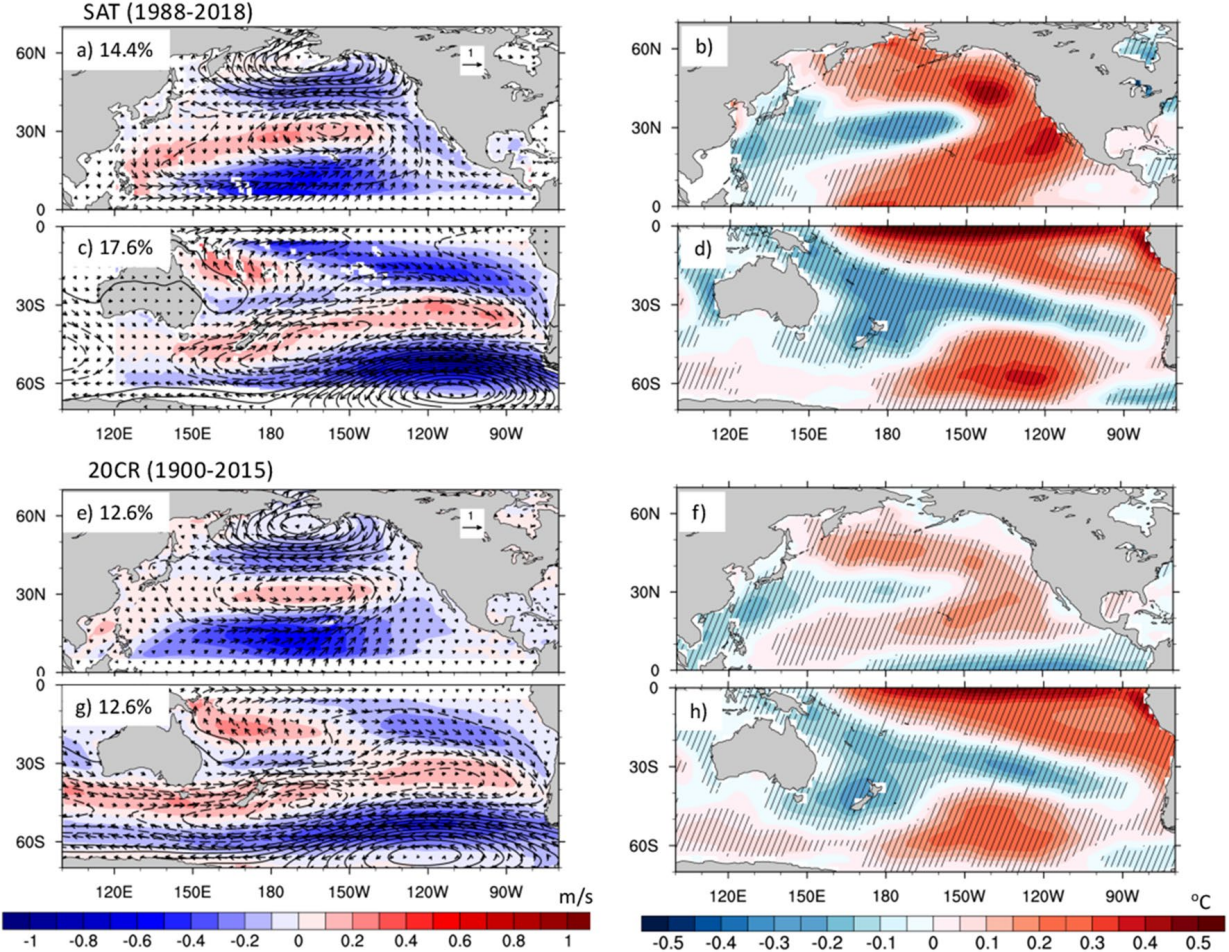
Hemispheric patterns of wind speed variability and associated SST anomalies. Leading EOFs of wind speed over the Northern (5° N–70° N) and Southern (5° S–70° S) Hemispheres for satellite winds (**a**, **c**) and 20CR-1900 (**e**, **g**). Numbers on the top-left corners indicate the variance explained by each EOF. Black contours and arrows show the linear regressions of SLP (in Pa) and vector wind (m/s) anomalies upon the corresponding Northern (**a**, **e**) and Southern (**c**, **g**) Hemispheres PCs for each dataset. Dashed contours indicate negative SLP values. Right panels show the linear regression of SST upon the wind speed PCs for satellite winds (**b**, **d**) and 20CR-1900 (**f**, **h**). Hatching shows regions with values statistically significant at the 95% level.

Thus, we repeat the EOF calculation in the two Hemispheres separately, using regions poleward of 5° to avoid the inclusion of equatorial wind signals. Results from the satellite merged winds are compared with those from 20CR-1900 in Fig. 2 (left panels). Similar results for ERA5 and 20CR-1988 are shown in Fig. S4. These hemispheric EOFs generally explain a larger fraction of variance than the basin-wide EOFs, especially for the satellite winds in the Southern Hemisphere (and ERA5 in the Northern Hemisphere, Fig. S4). Both satellites and 20CR-1900 show patterns of wind speed variability (and associated SLP and vector wind anomalies) in the Southern Hemisphere that are very similar to those found for the basin-wide EOFs. In both cases, we see a meridional SLP dipole typical of the PSA-1/SPO, and a reduction of the Southern Hemisphere trade winds. However, the Northern Hemisphere wind speed EOFs are now associated with SLP anomalies also characterized by meridional dipole patterns with poles centered around 50° and 60° N, respectively. This dipole pattern is consistent with the structure of the North Pacific Oscillation (NPO), the second leading pattern of wintertime SLP variability over the North Pacific^41,42^, whose equatorward pole is responsible for a weakening of the Northern Hemisphere trade winds during the phase displayed in Fig. 2a,e. The monthly standard deviations of the leading wind speed patterns in the Northern Hemisphere achieve their largest values in Winter and early Spring, while in the Southern Hemisphere the leading wind speed patterns exhibit their lowest standard deviation during January–March, with relatively small month-to-month variations during the rest of the years (Fig. S5), consistent with the findings of Lau et al.^38^ for PSA-1.

The SST anomalies (Fig. 2, right panels) associated with the wind speed leading patterns are obtained through linear regression on the standardized wind speed PCs. SST anomalies exhibit patterns similar to the Meridional Modes in both Hemispheres^12,14^ for both SAT and 20CR-1900. However, while the satellite winds show SST anomalies extending from Baja California to the central equatorial Pacific (160° E–130° W, Fig. 2b), 20CR-1900 displays subtropical SST anomalies that are more zonally oriented and reach the equator in the western part of the basin (Fig. 2f). Notice that 20CR-1988 shows a pattern very similar to that found for the satellite winds during the more recent period (Fig. S4), suggesting that the different pattern in 20CR-1900 may be due to a lack of stationarity of the winds-SST relationship over the twentieth century. These differences could also be partly related to uncertainties in the SST data used as boundary condition for 20CRv3 and to the more limited SLP observations prior to the satellite era. In the South Pacific, however, the SST patterns for satellites and 20CR-1900 are in very good agreement (Fig. 2d,h), and remarkably similar to the pattern known as the South Pacific Decadal Oscillation^43,44^. A similar agreement in the Southern Hemisphere is also found for ERA5 and 20CR-1988 (Fig. S4). These Southern Hemisphere SST patterns may also reflect the influence of the semi-annual oscillation (SAO)^45^, a twice-yearly meridional movement of the Southern Hemisphere SLP circumpolar trough, which has recently been related to the development of ENSO diversity^46^.

### Relationship with ENSO diversity

**Figure 3:**
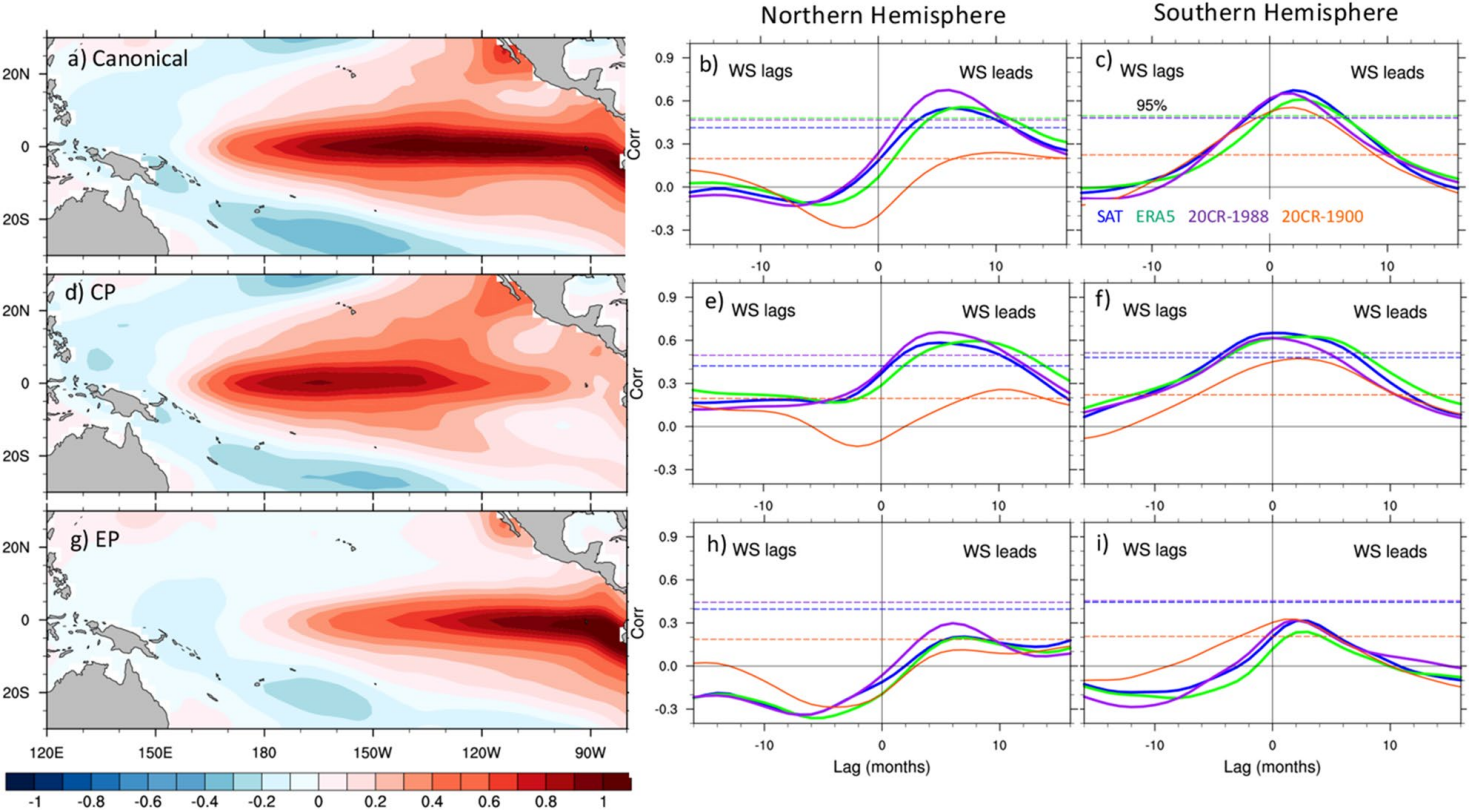
Flavors of El Niño diversity and their relationship with wind speed variability. (**a**) SST pattern associated with the leading PC of SST in the tropical Pacific (10° S–10° N), representing a “canonical” ENSO pattern. (**d**) and (**g**) CP and EP El Niño patterns obtained by regressing the SST field on the CP and EP indices (Eq. 2), respectively. The right two columns show the lag-correlations of the “canonical” (**b**, **c**), CP (**e**, **f**), and EP (**h**, **i**) indices with the Northern Hemisphere (middle column) and Southern Hemisphere (right column) wind speed PCs for satellite winds (blue), ERA5 (green), 20CR-1988 (purple), and 20CR-1900 (orange). Positive lags indicate that the wind speed PCs lead the ENSO indices. Horizontal dashed lines indicate the level of 95% statistical significance for the different products. The latter have been computed using the effective number of degrees of freedom (Eq. 1). Notice that the 95% level is much lower for 20CR-1900 (orange line) than for the other datasets due to the larger number of degrees of freedom over the longer record.

We have seen that the leading pattern of wind speed variability over the Pacific basin is well-correlated with a canonical ENSO index. Is that true also for the hemispheric EOFs and for different types of ENSO events? If so, are these wind anomalies more conducive to either EP or CP events? Do both Hemispheres have comparable influences, or does one dominate over the other? The patterns of canonical, CP and EP El Niño events are shown in the left panels of Fig. 3. While the Canonical El Niño type has large SST anomalies extending from the eastern ocean boundary to approximately the dateline, EP events exhibit their largest anomalies east of about 120° W, and CP events in the 170° E–130° W longitude range.

We explore the relationships between Pacific winds and ENSO types through lag-correlation analysis. The middle and right columns of Fig. 3 show the lag-correlation between the leading PCs of Northern and Southern Hemispheres wind speed, respectively, and the ENSO indices for all three wind products. The Northern Hemisphere winds show statistically significant correlations with both canonical and CP ENSO events for all three datasets after 1988. Peak correlations close to 0.6 are achieved when the winds lead the equatorial SST anomalies by about 6 months. However, correlations are much smaller, and barely significant, during the 1900–2015 period of 20CRv3. Statistically significant correlations are also found between the Southern Hemisphere winds and the canonical and CP ENSO types. In this case the largest correlations (~ 0.6–0.7) are achieved when the winds lead the canonical ENSO index by about 2–4 months, and the CP index by 0–5 months for the period after 1988, depending on the dataset. In the Southern Hemisphere, significant correlations are also found for 20CR-1900, suggesting a more robust influence of the Southern Hemisphere surface winds on these types of ENSO events over the century-long period.

Unlike canonical and CP ENSO events, the lag-correlation of the winds with the EP index are below the 95% statistical significance level for all datasets, except for 20CR-1900 over the Southern Hemisphere, which exhibits a marginally significant correlation, suggesting a much weaker relationship of the dominant wind patterns, and their associated SLP and SST anomalies, with events peaking in the far eastern Pacific. Inspection of the second WS EOFs does not indicate the existence of significant correlations of this second mode with any of the ENSO indices (not shown).

### Which basin-scale conditions are more conducive to EP and CP events?

**Figure 4:**
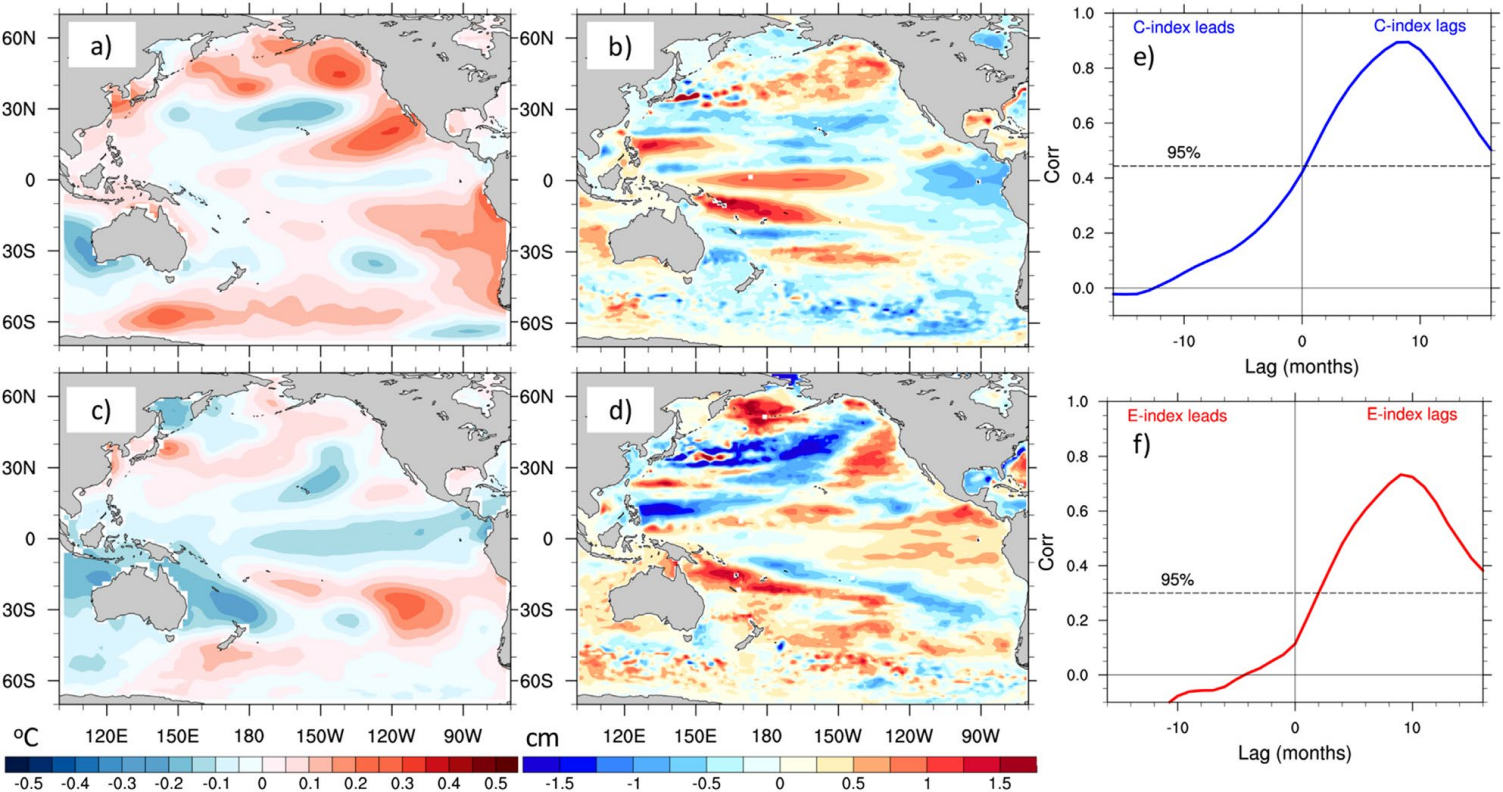
Basin-scale “sensitivity patterns” for CP and EP events. SST (**a**, **c**) and SSH (**b**, **d**) structures obtained by solving Eq. (3) for CP (top) and EP (bottom) ENSO events at a lead time *t* = 9 months. (**e**) Lag-correlation between the index associated with the SST/SSH sensitivity pattern for CP events and the CP index. Positive lags indicate that the sensitivity pattern index leads the CP index. The largest correlation (0.90) is achieved when the sensitivity pattern index leads the CP index by 9 months. (**f**) Same as (**e**), but for the EP index. In this case the largest correlation is 0.73, and is also achieved at a lead time of 9 months. Horizontal dashed lines show the 95% significant level in both cases.

If these leading patterns of wind speed do not have a significant relationship with EP events, are there different precursors, not captured by our wind speed modes, for EP events? To address this question, we consider the problem from an “inverse” perspective. Capotondi and Sardeshmukh^20^ used SST and SSH to identify precursors of ENSO diversity, and demonstrated the key role played by subsurface conditions, as encapsulated by SSH, in the selection of the ENSO type. Here, we extend that approach to the full Pacific basin (see “Methods” section), and then compute the associated wind speed, SLP, and precipitation fields through linear regression of those fields on the standardized indices of the SST and SSH precursor patterns. Equation 3 (see “Methods” section) is used to determine this optimal initial condition at a lead time *t* = 9 months. The initial SST and SSH fields that are most conducive to the mature CP and EP events are shown in Fig. 4a–d. In the case of CP events, the SST field includes structures similar to the PMM and the SPMM (Fig. 4a): in the Northern Hemisphere, SST anomalies extend from the coast of California to the central-western equatorial Pacific with decreasing amplitude, while in the Southern Hemisphere, anomalies are seen along the coast of South America, extending toward the eastern equatorial Pacific. Significant loading is also seen in areas along the North Pacific rim, with local maxima in the Gulf of Alaska, Bering Sea, and offshore of the Sea of Okhotsk, and in the South Pacific along 60° S. In the case of EP events (Fig. 4c), the largest positive SST anomalies are found along 20°–30° S, with a maximum around 120° W. Weak positive SST anomalies extending southwestward from the coast of California suggest a developing PMM, but their magnitude and extension seem too small to be impactful.

SSH anomalies for CP events (Fig. 4b) display a dipole pattern along the equator, with negative anomalies in the east, and positive anomalies in the central western Pacific, indicative of an enhanced zonal slope of the equatorial thermocline. On the contrary, EP events are characterized by positive SSH anomalies, and deeper thermocline, in the eastern Pacific (Fig. 4d), in agreement with previous studies^4,20^. SSH anomalies differences between EP and CP events are also seen in the extra-tropical Pacific. In particular, the SSH anomalies around 10°–15° N in the western Pacific, as well as the anomalies along the Kuroshio Extension and Gulf of Alaska, have opposite sign for the two event types. The SSH anomalies associated with CP events in the Northeast Pacific are somewhat reminiscent of the North Pacific Gyre Oscillation^47^, which was related to CP-type events at decadal timescales by Di Lorenzo et al.^48^.

A time series associated with the initial SST and SSH structures for each event type can be obtained by projecting the SST and SSH fields at each time step on those patterns. This is achieved by performing a scalar product, at each time step, between the state vector ***x ***containing the PCs of SST and SSH used for the analysis (see “Methods” sections) on the vector associated with the sensitivity pattern of either event type, which is also represented in the SST and SSH EOF space. This procedure is equivalent to a combined pattern correlation between the SST/SSH anomalies at any given time and the sensitivity patterns in Fig. 4. The lag-correlations of these time series with the corresponding CP and EP indices (Fig. 4e,f) show large and statistically significant correlations with maxima achieved when the time series of the “optimal” initial condition leads the CP and EP indices by 9 months.

**Figure 5:**
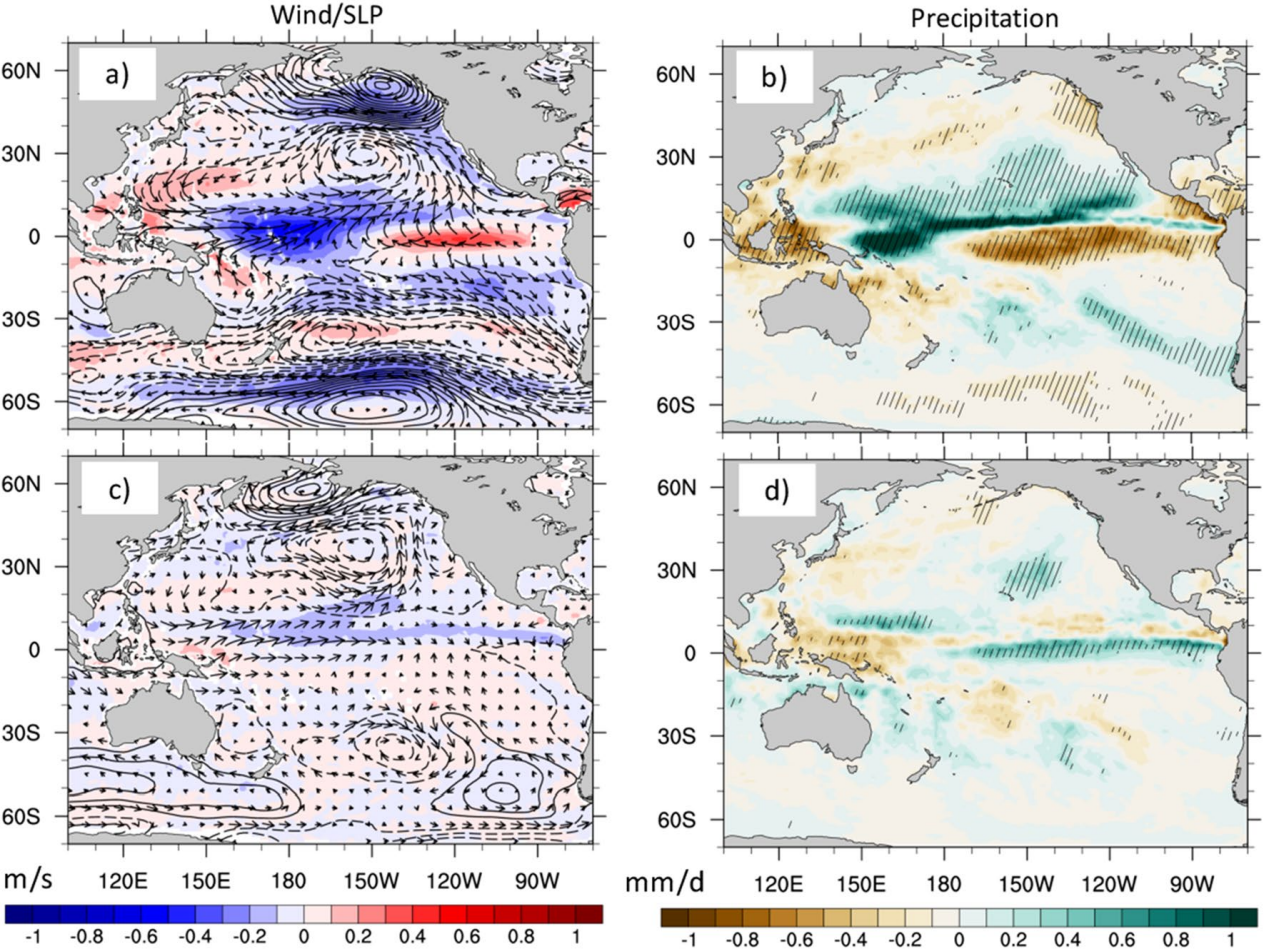
Atmospheric fields associated with the sensitivity patterns. (**a**) Linear regression of wind speed (shading, m/s), SLP (black contours, negative values dashed, Pa), and vector winds (arrows, m/s) on the index associated with the SST/SSH sensitivity pattern for CP events. (**b**) Linear regression of precipitation on the sensitivity pattern index for CP events. Hatched regions are statistically significant at the 95% level. (**c**) and (**d**). Same as (**a**) and (**b**), but for EP events. Wind speed data are from satellites, while SLP, vector winds and precipitation are from ERA5.

Having ascertained that the SST/SSH patterns in Fig. 4 can be viewed as precursors of CP and EP events, we use linear regression to determine the associated patterns of wind speed, vector winds, SLP and precipitation (Fig. 5). The patterns obtained for CP events are very similar to those identified through EOF analysis of WS (Fig. 2). In particular, we find SLP structures similar to the NPO and PSA-1/SPO in the Northern and Southern Hemispheres, respectively (Fig. 5a), together with their associated wind anomalies. Along the equator, westerly wind anomalies are seen in the western half of the basin, while easterly anomalies dominate in the eastern part of the basin. This wind pattern, which agrees with the observational findings of Harrison and Chiodi^49^, is consistent with the increased zonal thermocline tilt seen in Fig. 4b, which was considered an essential precursor of CP events by Capotondi and Sardeshmukh^20^. CP events are associated with a precipitation structure featuring enhanced precipitation in the far western Pacific and the Maritime continent and along a thin band just north of the equator, accompanied by reduced precipitation in the eastern equatorial Pacific, where the thermocline is shallower and SSTs are cooler.

The WS, SLP and vector wind regressions for the EP index (Fig. 5c) do not exhibit similarity with the patterns detected through EOF analysis, consistent with the lack of a statistically significant relationship between the EOFs of WS and the EP index. Indeed, the WS regressions are very weak everywhere, except for the equatorial region, where negative wind speed anomalies and the associated westerly wind anomalies extend all the way to the eastern Pacific. SLP anomalies are also relatively weak. In the Northern Hemisphere they display a meridional dipole resembling the NPO, while in the Southern Hemisphere, they are confined to high-latitudes and bear some resemblance with the pattern of PSA-2^38,39^. Precipitation anomalies associated with EP events display positive values extending from the dateline to the east Pacific, with negative values in the western part of the basin and just north of the equator, likely associated with an equatorward shift of the ITCZ. The wind and precipitation characteristics of EP and CP events identified by our analysis are consistent with those detected in a previous study^50^, where they were viewed as key discriminating features of moderate and extreme ENSO events.

## Summary and discussion

In this study, we use one satellite-based dataset and two state-of-the-art atmospheric reanalyses to characterize the leading patterns of wind speed variability over the Pacific basin through empirical orthogonal function (EOF) analysis. One of the reanalysis products extends back to the mid-1800s, allowing a comparison of wind variability over recent decades with those occurred over a century-long period. Pacific surface winds are a key component of most of the proposed extra-tropical ENSO precursors, as they promote formation of sea surface temperature (SST) anomalies through either thermo-dynamical or dynamical processes, but their specific role as ENSO precursors had never been previously investigated. Here we find that the dominant pattern of surface wind speed variability over the entire Pacific basin is similar among the different products and significantly correlated with ENSO variability at a lead time of 2–3 months. Locally, this pattern accounts for larger fractions of variance in some parts of the South Pacific and especially along the equator.

Hemispheric patterns of wind speed variability that exclude the equatorial band capture the leading SST and sea level pressure precursors proposed in the literature, including the Pacific Meridional Mode and North Pacific Oscillation in the Northern Hemisphere and the South Pacific Meridional Mode and South Pacific Oscillation in the Southern Hemisphere. These precursors are very similar among the three different datasets over the post-1988 period, but differences are detected in the structure of the Northern Hemisphere wind speed and SST precursors over the 1900–2015 period, suggesting a lower degree of stationarity of the Northern Hemisphere wind patterns prior to 1988. While these differences may be partly associated with a larger degree of sparseness and uncertainty in the data assimilated in the 20CRv3 reanalysis during the earlier part of the twentieth century, natural and/or anthropogenically-forced variations of the atmosphere–ocean system can also be expected to play an important role. ENSO characteristics, including the frequency of occurrence of EP and CP events, appear to vary on decadal timescales in both observations and long model simulations^51–53^. In addition, the linear detrending applied to all the variables used in this study is likely inadequate to remove the climate change signal, which may be more pronounced in recent decades and may also manifest itself in the form of low-frequency decadal variations^54^ rather than a linear trend. A study based on the Community Earth System Model (CESM) Large Ensemble^55^ detected a strengthening of the North Pacific Meridional Mode with climate change, which could explain a more prominent influence of the Northern Hemisphere on ENSO in the recent period. Disentangling these issues is beyond the scope of the present study, but an in-depth analysis of low-frequency ENSO variations using the century-long atmosphere and coupled reanalyses that are becoming available may illuminate the evolving nature of ENSO precursors, and will be considered in future studies. It will also be important to repeat similar analyses in climate models that provide a “realistic” simulation of ENSO diversity, using long model simulations to verify the present results with a higher degree of statistical significance, and scenario simulations to examine the influence of climate change on ENSO precursors and their relationship with ENSO diversity.

The leading patterns of wind speed variability are significantly correlated with “canonical” and CP-type ENSO events after 1988, but correlations for the Northern Hemisphere patterns are barely significant over the century-long period, again suggesting a possibly less robust role for Northern Hemisphere wind precursors, with the caveats noted above. Patterns of SST and SSH similar to those detected with this analysis also emerge as optimal precursors of canonical ENSO events in a millennium-long integration of the National Center for atmospheric Research (NCAR) Community Earth System Model version 2 (CESM2)^56^, implying a relevant role for both Hemispheres in the model context, and suggesting the need for further investigations of the relative role of Northern and Southern Hemisphere precursors in observations.

A novel result of this study is that the patterns of Northern and Southern Hemisphere wind speed variability show no statistically significant correlation with EP ENSO events during the satellite era. While the post-1988 period was predominantly characterized by CP ENSO events, the results obtained using the 20CRv3 reanalysis over the 1900–2015 period also show correlations of the wind patterns with EP events that are statistically insignificant or only marginally significant, supporting the results obtained during the satellite era. To gain insight in the nature of EP precursors, we use the statistical approach of Capotondi and Sardeshmukh^20^ to determine the SST and sea surface height (SSH) structures that are most conducive to either EP or CP event types three seasons later, and then determine the associated wind, sea level pressure and precipitation patterns through linear regression. The indices associated with these SST and SSH initial conditions for each event type appear to be well correlated with the EP and CP indices at the later time. The wind speed and sea level pressure fields obtained with this approach are very similar to those obtained through EOF analysis for CP events, confirming the key role played by extra-tropical SLP and wind precursors for this type of events. The main wind anomalies for EP events are found along the equator, and are associated with a penetration of the anomalous equatorial westerlies to the eastern part of the basin.

We note that the indices used to characterize ENSO diversity emphasize the distinction between moderate (CP) and extreme (EP) event types^57^. Our results confirm that the CP events detected with this approach capture the larger cluster of moderate events discussed in Takahashi and Dewitte^57^. These events appear to be related to well-known extra-tropical precursors, and their exact longitudinal location and spatial pattern may be modulated by the initial zonal thermocline slope. As in Takahashi and Dewitte^57^, EP events are rarer and appear to be associated with conditions that allow the equatorial wind (and convection/precipitation) anomalies to extend to the eastern Pacific. These conditions may occur during positive phases of the Pacific Decadal Oscillation, which is associated with a reduced zonal thermocline slope^58^ and may be favored by enhanced stochastic wind activity in the form of Westerly Wind Events^59^. Influences from other oceans, as recently suggested for the Atlantic^60^, may also play a role.

This study has demonstrated that surface wind speed data from satellite retrievals are now of sufficient duration and accuracy to be suitable for climate studies, and may provide an excellent benchmark for reanalysis products that do not assimilate them (e.g., 20CRv3). The continuation of satellite missions, together with sustained in situ wind observations for calibration and validation of the satellite retrievals, will be of paramount importance for an increasingly robust assessment of ENSO wind forcing in the context of a changing climate.

## Supplementary Information

Supplementary Figures.

## Data Availability

The satellite wind speed data used in this study are available at http://www.remss.com/measurements/wind/wspd-1-deg-product/. The 20CRv3 dataset can be downloaded from https://psl.noaa.gov/data/gridded/data.20thC_ReanV3.html. The ERSSTv5 is from https://psl.noaa.gov/data/gridded/data.noaa.ersst.v5.html. The output of the ECMWF ERA5 atmospheric reanalysis is available at https://www.ecmwf.int/en/forecasts/dataset/ecmwf-reanalysis-v5, and the ECMWF ORAS5 can be obtained from https://www.ecmwf.int/en/forecasts/dataset/ocean-reanalysis-system-5. The sensitivity patterns output can be obtained from the corresponding author.
