# Supplementary material for: The influence of pacific winds on ENSO diversity
Source: Sci Rep. 2021 Sep 21;11:18672. doi: 10.1038/s41598-021-97963-4 (PMC8455613; doi:10.1038/s41598-021-97963-4)
Supplement: Supplementary file 1 — Supplementary Figures. [file 41598_2021_97963_MOESM1_ESM.pdf]

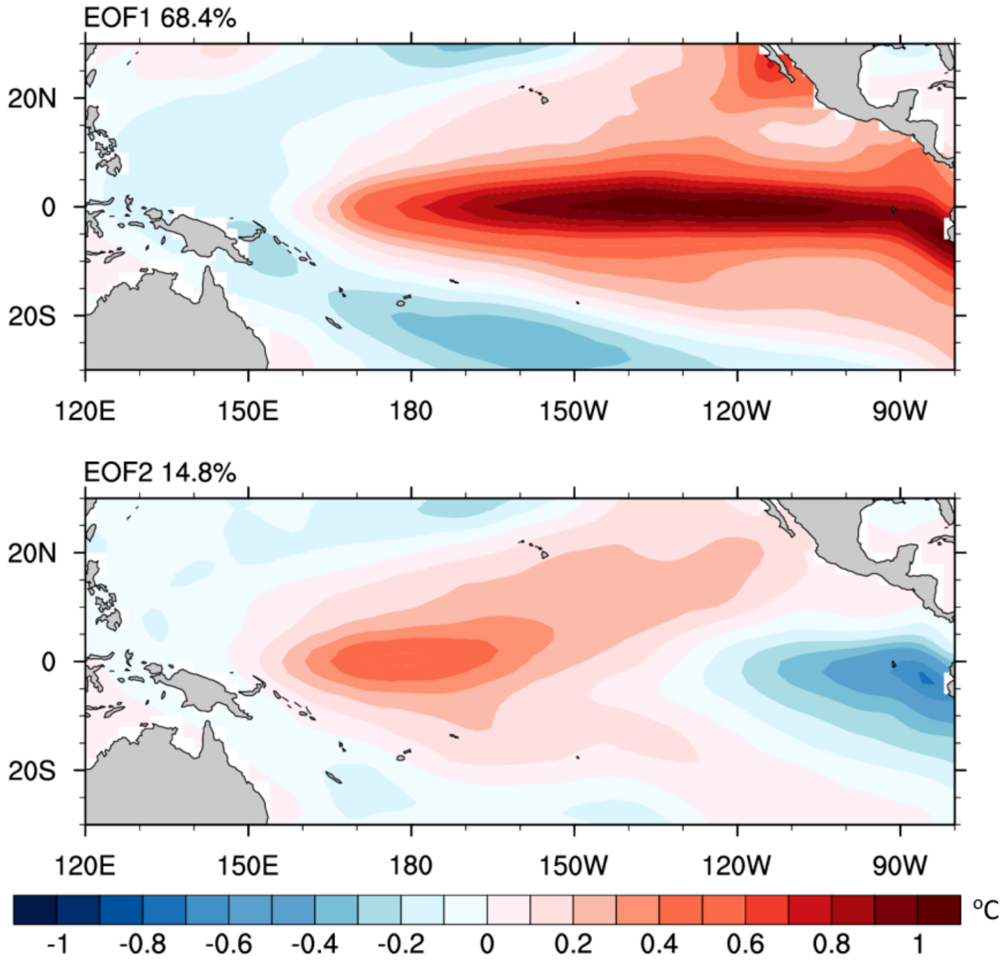

Figure S1. Leading patterns of tropical Pacific SST. SST anomalies associated with the first (top) and second (bottom) EOF patterns of equatorial Pacific (10ºS-10ºN) SSTs. These two EOFs accounts for 68.4% and 14.8% of the SST variance in the equatorial band. EOF1 describes a pattern similar to that of a "canonical" El Niño event, while EOF2 displays an equatorial Pacific dipole with anomalies of one sign in the far eastern Pacific, and anomalies of the opposite sign in the central Pacific. The linear combination of these two patterns (equation 2) produces EP and CP event types.

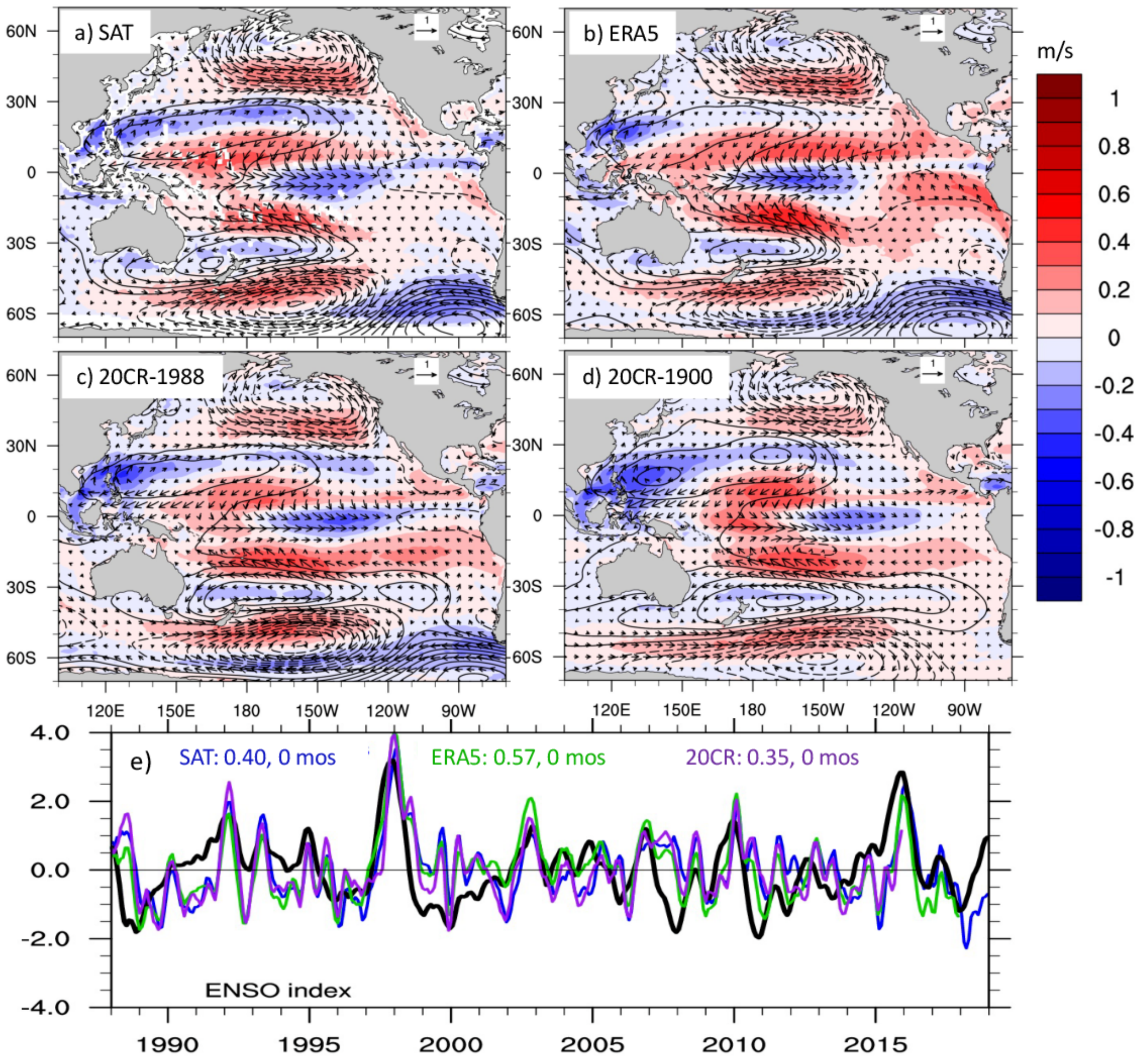

Figure S2. Pacific patterns of wind speed variability and relationship with ENSO. Second EOF of wind speed for a) Merged satellite winds (variance explained 6.8%), b) ERA5 (8.3%), c) 20CRv3 during 1988-2015 (7.5%), and d) 20CRv3 over 1900-2015 (6.9%) over the Pacific basin (70ºS-70ºN, 100ºE-70ºW). Black contours show the regression of SLP anomalies (in Pa) upon the EOFs' time series, or Principal Components (PCs). Dashed contours indicate negative values. Arrows show the vector winds regressed on the leading PCs of each wind speed product. SLP and winds from ERA5 are used in a) and b), while SLP and winds from 20CRv3 are used in c) and d). Vector winds are in m/s. e) Comparison between the canonical ENSO index (black, see Methods), and the PCs of the second wind speed EOFs, as indicated by the different colors. Numbers on the top indicate the maximum correlation and the wind speed lead time at which the maximum correlation is achieved for each data set, as indicated by the different colors. The largest correlations are achieved at lag 0, and are below the 95% statistical significance level for SAT and 20CR (0.47 and 0.46, respectively), and slightly above that significance level (0.5) for ERA5.

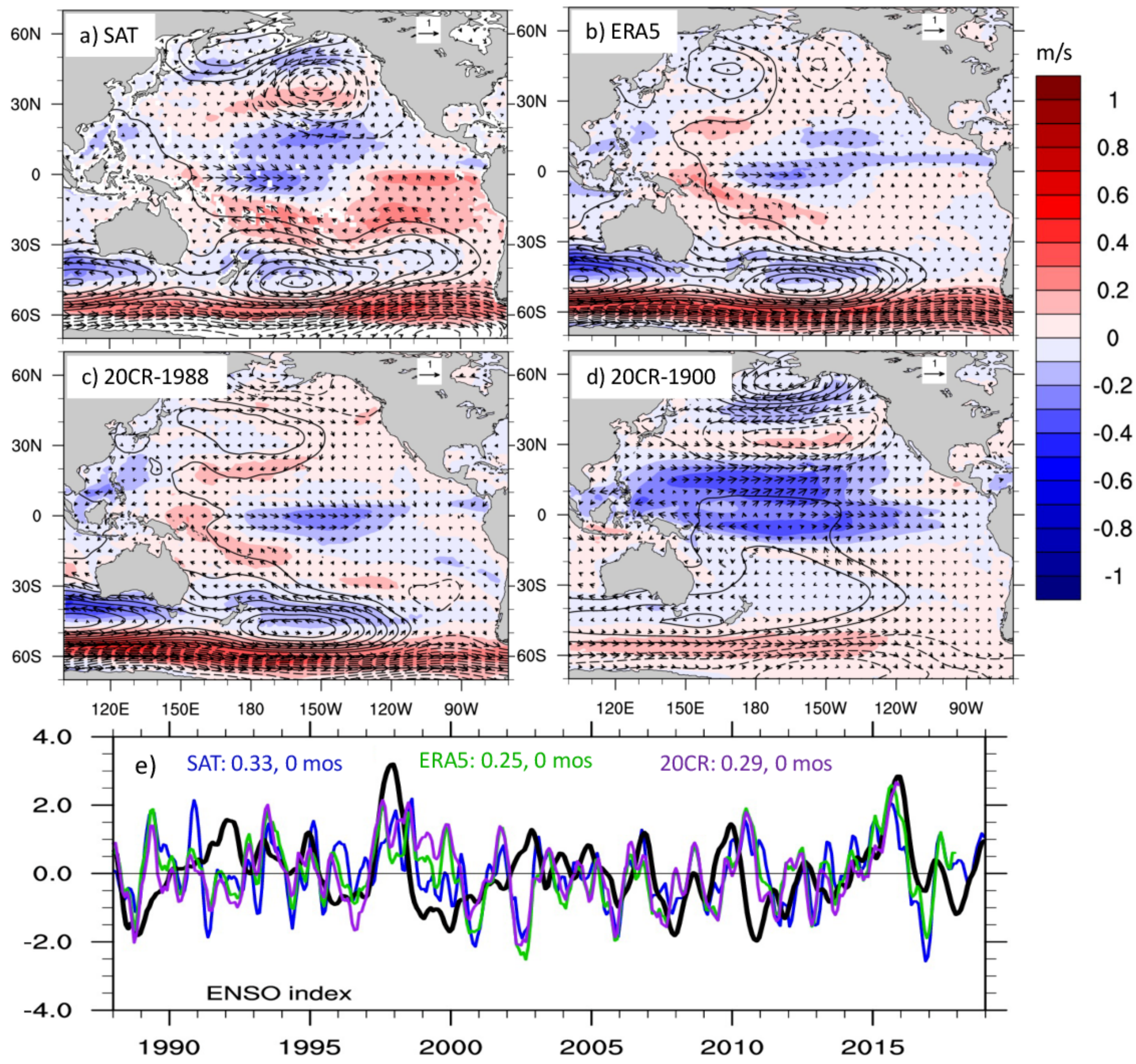

Figure S3. Pacific patterns of wind speed variability and relationship with ENSO. Third EOF of wind speed for a) Merged satellite winds (variance explained 5.6%), b) ERA5 (6.1%), c) 20CRv3 during 1988-2015 (6.6%), and d) 20CRv3 over 1900-2015 (6.6%) over the Pacific basin (70ºS-70ºN, 100ºE-70ºW). Black contours show the regression of SLP anomalies (in Pa) upon the EOFs' time series, or Principal Components (PCs). Dashed contours indicate negative values. Arrows show the vector winds regressed on the leading PCs of each wind speed product. SLP and winds from ERA5 are used in a) and b), while SLP and winds from 20CRv3 are used in c) and d). Vector winds are in m/s. e) Comparison between the canonical ENSO index (black, see Methods), and the PCs of the third wind speed EOFs, as indicated by the different colors. Numbers on the top indicate the maximum correlation and the wind speed lead time at which the maximum correlation is achieved for each data set. The largest correlations are achieved at lag 0, and are below the 95% significance level (0.40, 0.46, and 0.46 for SAT, ERA5 and 20CR, respectively).

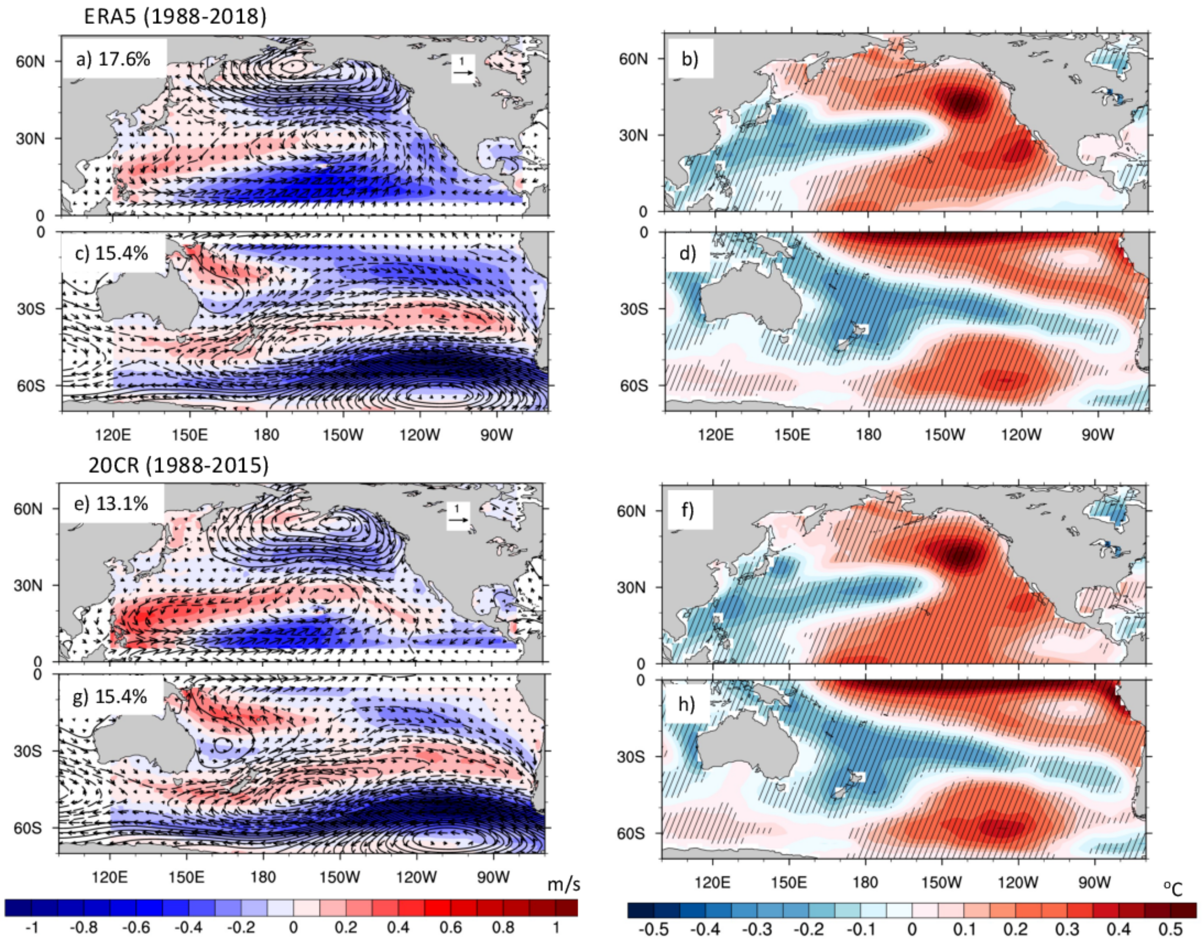

Figure S4. Hemispheric patterns of wind speed variability and associated SST anomalies. Leading EOFs of wind speed over the Northern (5ºN-70ºN) and Southern (5ºS-70ºS) Hemispheres for ERA5 (a, c) and 20CR-1988 (e, g). Numbers on the top-left corners indicate the variance explained by each EOF. Black contours and arrows show the linear regressions of SLP (in Pa) and vector wind (m/s) anomalies upon the corresponding Northern (a, e) and Southern (c, g) Hemispheres PCs for each dataset. Dashed contours indicate negative SLP values. Right panels show the linear regression of SST upon the wind speed PCs for ERA5 (b, d) and 20CR-1988 (f, h). Hatching shows regions with values statistically significant at the 95% level.

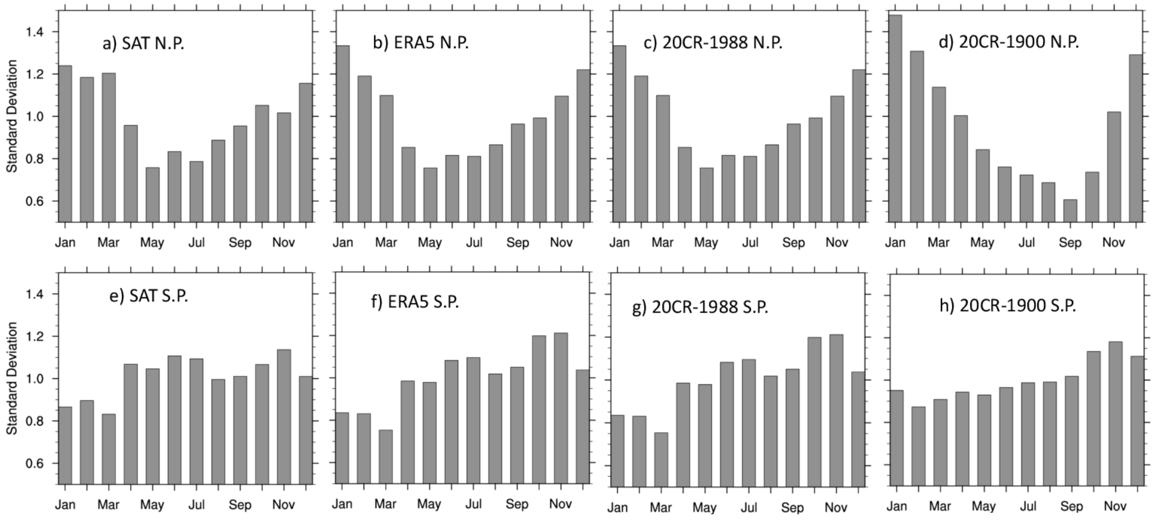

Figure S5. Seasonality of wind speed patterns. Monthly standard deviations of the PCs associated with the leading modes of wind speed variability over the Northern Hemisphere (top row) and Southern Hemisphere (bottom row) for satellites (a, e), ERA5 (b, f), 20CR-1988 (c, g), and 20CR-1900 (d, h).
